# Data supporting the nuclear phylogenomics of the palm subfamily Arecoideae (Arecaceae)

**DOI:** 10.1016/j.dib.2016.02.063

**Published:** 2016-03-02

**Authors:** Jason R. Comer, Wendy B. Zomlefer, Craig F. Barrett, Dennis Wm. Stevenson, Karolina Heyduk, James H. Leebens-Mack

**Affiliations:** a University of Georgia, Department of Plant Biology, Athens, GA 30602−7271, USA; b California State University, Los Angeles, Department of Biological Sciences, Los Angeles, CA 90032−8201, USA; c New York Botanical Garden, Bronx, NY 10458−5126, USA

**Keywords:** Ancestral area, Arecaceae, Arecoideae, Coalescent, Nuclear phylogeny, Targeted sequencing

## Abstract

This data article provides data and supplemental materials referenced in “Nuclear phylogenomics of the palm subfamily Arecoideae (Arecaceae)” (Comer et al., 2016) [1]. Raw sequence reads generated for this study are available through the Sequence Read Archive (SRA Study Accession: SRP061467). An aligned supermatrix of 168 nuclear genes for 35 taxa (34 palms and one outgroup taxon) is provided. Also provided are individual maximum likelihood gene trees used for the coalescent based analyses, output from the maximum parsimony analyses, and two figures.

**Specifications Table**

**Table:** 

Subject area	Biology, Genetics and Genomics
More specific subject area	Phylogenetics and Phylogenomics
Type of data	*Sequence alignment, analysis output file, and figures*
How data was acquired	*Hybrid gene capture and Illumina MiSeq sequencing.*
Data format	*Raw and analyzed.*
Experimental factors	*Hybrid gene capture on total genomic DNA, following the protocol of Heyduk et al.*[2]*and Comer et al.*[3].
Experimental features	*Following hybridization and sequencing, 168 nuclear genes (for 35 taxa) were used for phylogenetic analyses.*
Data source location	*Newly sampled taxa for this dataset were collected from Cameroon, Florida, Ghana, and Thailand. See also*Appendix A*in Comer et al.*[1].
Data accessibility	*Data is within this article. For raw sequence reads see SRA Study Accession: SRP061467.*

**Value of the data**

• Provides a dataset of 168 nuclear genes for 34 palm taxa and one outgroup taxon.
• Provides a nuclear phylogeny for the palm family from the largest dataset to date.
• Provides a foundational dataset for future phylogenomic studies of palms.

## 1 Data

**Fig. 1:**
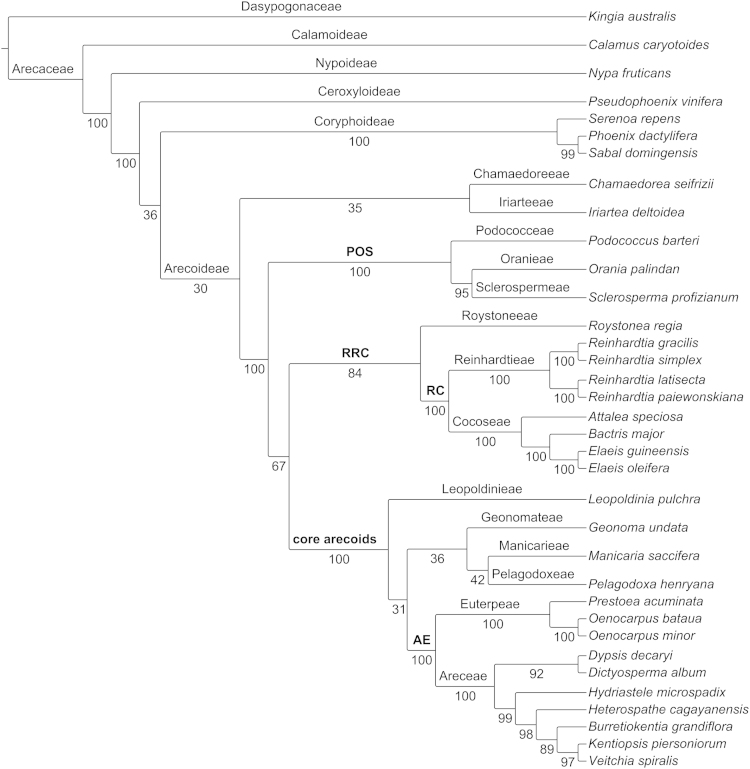
Species tree (most parsimonious) from the MP concatenated analysis of the 168 nuclear genes. Labels above the branches=family, subfamily, tribe, and major clade (boldface font); labels below branches=bootstrap support. Major clades: AE (Areceae+Euterpeae), core arecoids (Areceae, Euterpeae, Geonomateae, Leopoldinieae, Manicarieae, and Pelagodoxeae), POS (Podococceae, Oranieae, and Sclerospermeae), RC (Reinhardtieae+Cocoseae), and RRC (Roystoneeae, Reinhardtieae, and Cocoseae).

**Fig. 2:**
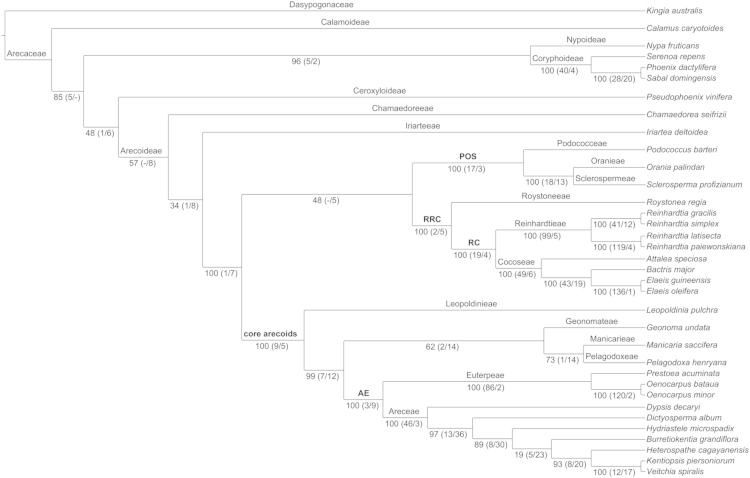
Species tree from the ASTRAL analysis of the best gene trees of the 168 nuclear genes. Labels above the branches=family, subfamily, tribe, and major clade (boldface font); labels below branches=bootstrap support; numbers in parentheses=gene trees supporting (monophyletic) or rejecting (polyphyletic) the clade with a bootstrap value≥75; a dash (–) indicates no genes trees with a bootstrap value of≥75. Major clades: AE (Areceae+Euterpeae), core arecoids (Areceae, Euterpeae, Geonomateae, Leopoldinieae, Manicarieae, and Pelagodoxeae), POS (Podococceae, Oranieae, and Sclerospermeae), RC (Reinhardtieae+Cocoseae), and RRC (Roystoneeae, Reinhardtieae, and Cocoseae).

The dataset shared here consists of the 168 aligned nuclear gene supermatrix (Supplementary material 1) used in Comer et al. [1]. Also shared within this article are supporting material referenced in Comer et al. [1] (Supplementary material 2–4 and Fig. 1, Fig. 2).

## 2 Experimental design, materials and methods

### 2.1 Taxon sampling and hybrid gene capture

Thirty-four species were sampled, representing the five palm subfamilies and the 14 tribes of subfamily Arecoideae (see Comer et al. [1] Appendix A). Total genomic DNA was sheared with a Covaris sonicator (Woburn, MA, USA) to an appropriate size then used for Illumina library construction (see also Comer et al. [1], [3] and Heyduk et al. [2]). Resulting genomic libraries were enriched for target nuclear exons through hybridization to RNA baits (MYcoarray, Ann Arbor, Michigan, USA) [2], [3], [4]. Hybridization reactions were pooled for paired-end sequencing on the Illumina MiSeq platform [3].

### 2.2 Assembly

Sequence reads were demultiplexed, quality trimmed from the 3′ ends, and filtered [1], [2], [3]. The *de novo* assembler Trinity v. 2.06 [5] was used to assemble the cleaned reads, and CAP3 v. 102011 [6] was used to collapse assembled contigs [1]. Assembled contigs with segments matching the target exons were identified using BLAST (Basic Local Alignment Search Tool; Expect value 1×10^−20^; [7]). Following Heyduk et al. [2], duplicate contigs were removed to reduce the potential for paralogy (see Fig. 2b in Comer et al. [1]). Exons from the same gene were concatenated into super scaffolds. For summary statistics see Table 2 in Comer et al. [1].

Assembled genes were aligned using PRANK v. 100802 [8], and Gblocks v. 0.91b [9] was used to filter poorly aligned and non-conserved regions [1]. Genes were excluded if a significant amount of data was missing or if the aligned gene exhibited an average pair-wise genetic distance of more than 0.15 [1]. Scripts used for this study’s assembly pipeline can be found at: https://github.com/kheyduk/reads2trees.

### 2.3 Phylogenetic reconstruction

Phylogenetic analyses were performed using supermatrix and coalescence-based species tree estimation approaches utilizing the 168 nuclear gene dataset presented here (Supplementary material 1). For the maximum parsimony, aligned genes were concatenated into a single supermatrix alignment (Supplementary material 1) and the TNT v. 1.1 (Tree Analysis Using New Technology, Willi Hennig Society edition; [10], [11]) “one-shot” analysis script (consecutively ran random addition sequences, TBR, sectorial searches, and tree fusing each iteration for 20 iterations, 100 random addition replications and 1000 standard bootstrap replicates) was used for phylogenetic reconstruction (Fig. 1 and Supplementary material 2). ASTRAL v. 4.7.8, a coalescent based species tree estimation method, was used to estimate the species tree [12] from individual gene trees and bootstrap replicates estimated with RAxML (GTRGAMMA, ‘-f a’, and 500 bootstrap replicates; Supplementary material 3 and 4) [13], [14], [15]. We used the ASTRAL’s heuristic version to implement a multi-locus bootstrapping analysis for both the ML best scoring gene trees (Fig. 2) and the ML bootstrap replicates (Fig. 3 in Comer et al. [1]).

## Footnotes

Appendix A Supplementary data associated with this article can be found in the online version at doi:10.1016/j.dib.2016.02.063.
