# Supplementary material for: Data supporting the nuclear phylogenomics of the palm subfamily Arecoideae (Arecaceae)
Source: Data Brief. 2016 Mar 2;7:532–6. doi: 10.1016/j.dib.2016.02.063 (PMC4796708; doi:10.1016/j.dib.2016.02.063)
Supplement: Supplementary file 1 — Supplementary material [file mmc1.docx]

Conflict of interest: none
